# Basic Behavioral Processes Involved in Procrastination

**DOI:** 10.3389/fpsyg.2021.769928

**Published:** 2021-11-23

**Authors:** Thomas R. Zentall

**Affiliations:** Department of Psychology, University of Kentucky, Lexington, KY, United States

**Keywords:** procrastination, delay discounting, delay reduction theory, Sidman avoidance, negative reinforcement

## Abstract

Procrastination involves an irrational putting off of engaging in a course of action, in spite of expecting to be worse off for the delay. I suggest that to understand the processes underlying procrastination one should examine its relation to several behavioral procedures that have been studied in humans and other animals. For example, in delay discounting, smaller rewards that come sooner are often preferred over larger rewards that come later. In the context of delay discounting, procrastination can be viewed as the preference for an immediate competing activity over the delay to work on a required task. Another process similar to procrastination can be seen in free operant, temporal avoidance (or Sidman avoidance) in which an animal will receive a shock (a deadline not met) if an interval passes without a specified response (task completion). Once animals learn about the interval, they often procrastinate by waiting until the interval has almost passed before responding. Finally, research with animals suggests that the persistence of procrastination may involve a form of negative reinforcement associated with the sudden decline in anxiety or fear (relief) when the task is completed prior to the deadline. Research with animals suggests that the mechanisms responsible for human procrastination may involve systems that derive from several procedures known to produce similar behavior animals.

## Introduction

Comparativepsychology has a longhistory of providing models relevant to human behavior. For example, there is considerable evidence that Pavlovian conditioning procedures are relevant to the conditioning of human emotional response (fear conditioning, taste and odor conditioning, phobias). Similarly, procedures derived from behavior analysis have been found to be relevant to the training of special needs children to care for themselves. They can respond to alternatives in direct proportion to the distribution of reinforcements (the matching law; Herrnstein, 1961; Baum, 1974). Even in the context of complex human decision making, comparative research has demonstrated the relevance of how animals make decisions (Zentall and Wasserman, 2012). Comparative research can provide us with hypotheses concerning the evolution and biological basis of human behavior. Comparative research can often contribute to our understanding of behavior because the learning processes are often simpler in animals than in humans, and we can better control the prior learning experiences. Research with animals does have the drawback that we cannot use their self-report to explain the basis for their choice, so that has to be inferred.

The purpose of the present article is to examine the comparative psychological research for phenomena that might clarify the mechanisms responsible for the tendency for humans to procrastinate. I will start with a brief description of the mechanisms thought to be responsible for human procrastination. Although procrastination is typically viewed as a uniquely human phenomenon, the results of several, well-studied procedures with animals suggest that the mechanisms involved in procrastination may be closely related to the behavior that one sees in other animals (see Zentall, 2020). With this in mind, I will identify three lines of animal research that suggest they are related to human procrastination. The first, delay discounting is the idea that small immediate rewards are often preferred over larger delayed rewards. Steel and König (2006) have proposed that temporal motivation theory, based on delay discounting, to be the mechanism responsible for human procrastination (see also Steel, 2007). According to this theory, completion of the required task represents the larger delayed reward and competing activities represent the smaller more immediate rewards.

The second line of research is free operant, temporal avoidance, a procedure in which an aversive event is signaled only by the passage of time (Sidman, 1966). It is a procedure that Zentall (2020) identified as analogous to human procrastination in which an animal will receive a shock if it does not make a required response within a defined interval of time.

The third mechanism is negative reinforcement, the idea that the removal of an aversive event can be reinforcing. If completion of the required task relives anxiety it can provide an additional incentive for repeated procrastination. To the extent that these mechanisms are involved in human procrastination, understanding them may allow for a better appreciation for why we procrastinate and potentially, what we can do to reduce its negative effects.

## Procrastination by Humans

Procrastination is often viewed as a trait (e.g., Arvey et al., 2003), and generally a somewhat negative trait (Schouwenburg, 2004). However, it is more realistically viewed as a graded continuum that is affected by many contextual and experiential variables. One way to view procrastination is the balance between the aversiveness of the approaching deadline, together with the anxiety associated with missing the deadline, vs. the attractiveness of alternative activities (e.g., watching television or socializing). Viewed from this perspective, procrastination is typically the choice between an immediate positive activity and the delayed avoidance of a negative outcome (missing a deadline).

Research with animals comes with an inherent challenge, one is limited to their motor behavior because they cannot report how they feel about their choices. On the other hand, what people say about their behavior may not always reflect their underlying motivation. Often humans do not know why they make their choices nor how they feel about them afterwards. Focus on the behavior of animals strips the behavior down to basic principles and allows us to observe the behavior in the absence of human cultural effects. It also allows us to consider the possibility that in the context in which the behavior evolved, the behavior may not be as irrational as it may first appear.

It is well known that humans function using two different systems. One involves implicit (automatic), fast subconscious processes such as procedural, skill, and habit learning that are associated with brain activity in the basal ganglia (Mishkin et al., 1984). The other involves an explicit (controlled), slow conscious process such as executive attention, working memory hypothesis testing and rule formation that are associated with brain activity in the prefrontal cortex, the anterior cingulate gyrus, the head of the caudate nucleus, and the hippocampus (Fuster, 1989). In humans, it is often difficult to separate the implicit and explicit processes that may be involved in procrastination. To the degree that we do not have easy access to the implicit processes that may be present in human procrastination, we may be able to learn about them from studying other animals.

## The Explicit Study of Procrastination in Animals

**Figure 1:**
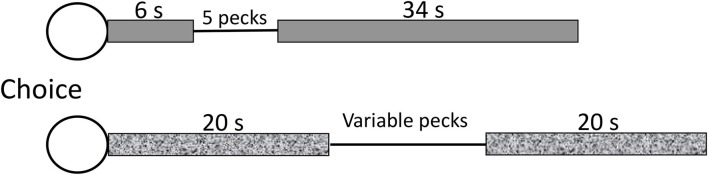
After Mazur (1996). Pigeons were fed during the timed intervals on a variable time schedule (response independent). The variable response requirement was titrated until the pigeon was indifferent between the two chains. The measure of procrastination was the number of pecks required (>5) for the pigeons to be indifferent between the two chains.

Surprisingly, there has been very little research with animals directed to the study of procrastination itself. This may be because it is difficult to imagine an aversive task for an animal, such as writing a term paper for a human, that can be delayed but not omitted. One of the only studies on animal procrastination was conducted with pigeons (Mazur, 1996). In that experiment, pigeons were given a choice between completing a peck requirement early and completing a peck requirement late. Mazur's procedure was relatively complex so I will go over it. He started with a procedure that looks somewhat like delay discounting. He gave pigeons a choice between making 5 pecks after 6 s or making more than 5 pecks after 20 s (see Figure 1). He asked, how many pecks would it take after 20 s for the pigeon to be indifferent between the sooner 5-peck requirement and the deferred increased peck requirement. He found that the pigeons chose to make up to 30 pecks if they could defer pecking for as little as 14 s (the difference between 20 and 6 s).

To further complicate matters, in Mazur's procedure, the time before the peck requirement (as well as after the peck requirement) was signaled by a houselight and during those intervals, intermittent food was provided on a variable time schedule (occasionally, food was made available independent of pecking). The intermittent food presented before engaging in the pecking task can be thought of as humans engaging in pleasurable activities and it is clear that the pigeons preferred to delay engaging in the pecking because they were willing to peck six times as much if they could defer the pecking task. In effect, what this means is that when pecking was required, it represented a period during which reinforcement could not be obtained. Mazur found that the pigeons preferred delaying the nonreinforced peck requirement. They preferred delaying the pecking, likely because the peck requirement interrupted the food-associated houselight cue. Consequently, in addition to deferring the pecking requirement, it is likely that the pigeons preferred to extend the conditioned stimulus (houselight) during which time they could receive food. Extending the conditioned stimulus (houselight) can be thought of as similar to humans choosing to engage in a pleasurable activity such as socializing with friends or watching television, rather than working on a term paper.

## Procrastination and Delay Discounting

When one procrastinates, one is often choosing to delay an aversive activity to engage in a pleasant or less aversive activity. Steel and König (2006) present a theory of procrastination called temporal motivation theory. This theory is based on several human parameters but most notable is the discounting of the value of a future outcome, as a function of the time (or delay) to that outcome. Thus, procrastination can be viewed in the broader context of delay discounting. Delay discounting is generally applied to contexts in which the choice is between a smaller reward sooner and a larger reward later (Ainslie, 1975). Depending on the nature of the reward, the amounts of the smaller and larger rewards, and the delay between the shorter and longer reward, one often finds that there is a smaller sooner reward that will be preferred over the larger later reward. For example, a person might prefer an offer of $5 now over $10 next week.

Research on delay discounting of rewards suggests that humans (and other animals) often make decisions to select a smaller reward sooner rather than a larger reward later, decisions that that are usually considered to be suboptimal (e.g., Odum, 2011). The decisions are suboptimal in the sense that the amount of reward obtained is less than would be obtained had they waited for the larger reward.

Although some have suggested that procrastination, choice of the smaller sooner, involves the irrational putting off of a task (Silver and Sabini, 1981; Akerlof, 1991), in the context of delay discounting, at the time the decision is made, it may not be considered irrational by the decision maker. Even if an individual intends not to procrastinate in advance, and possibly later regrets having procrastinated, it does not alter the fact that at the time the decision to procrastinate was made, it may have been a rational decision—a choice between two alternatives, one that had a greater value to the individual *at that time* than the other alternative.

For humans, when it comes to purchasing things, if one does not have the money, one may charge the items to a credit card, rather than waiting until enough money is saved to buy the items with cash. If the items represent something the individual wants but perhaps does not need, some people would consider these purchases to be irrational. However, choosing the smaller sooner is not always an irrational choice. For example, it is quite rare to wait until one has accumulated enough money to purchase a house. Instead, it is considered quite rational to save enough for a down-payment and then obtain a mortgage for the rest, even if taking on a mortgage means paying much more for the privilege of living in the house sooner. In both of these cases waiting would be aversive, but most people would consider taking out a mortgage for the purchase of a house to be a rational decision, whereas some might consider the purchase of less necessary items to be able to enjoy them right away to be an impulsive irrational choice. Thus, in some cases, it would be considered quite rational to incur the greater cost to obtain the benefit sooner.

One could argue that in the case of procrastination the choice does not involve a deferred reward but a deferred somewhat aversive task. Thus, perhaps a better animal analog to human procrastination is one involving the deferring of an aversive event. Such a procedure was used by Liley et al. (2019). They gave rats a choice between two levers, one that provided one pellet of food and the other that provided three pellets of food. If the rats chose the three-pellet lever, however, they also received a foot shock (analogous to having to complete an aversive task). Although for the rats, the pellets were delivered immediately, the shock was delivered after a delay. What they found was the more delayed the shock, the more preferred was the three-pellet alternative. If one considers the shock to be analogous to an impending deadline, the further off the deadline, the more likely the rats were to choose the more reinforcing three-pellet alternative. That is, when the deadline (shock) was more imminent, the rats chose to avoid the more favorable three-pellet reward with its accompanying penalty (fear of shock) in favor of the less favorable one-pellet reward.

Delay discounting and procrastination both involve the choice between a more immediate positive event and a more delayed alternative event. In the case of delay discounting, the immediate and delayed events are typically appetitive. In the case of procrastination, the immediate event may be appetitive (e.g., watching TV) or aversive (e.g., washing dishes), but the immediate event is usually less aversive than the delayed event (e.g., writing a term paper).

## The Power of the Hyperbolic Function of Delay Discounting

Some have proposed that it is considered procrastination only when delaying the start of the task is unintentional (Silver and Sabini, 1981; Lay and Silverman, 1996). That is, if one intentionally delays the start of the task it would be considered an informed planned decision, unlikely to create anxiety. One can argue, however, that procrastination is always intentional, at least it is *at the time* one *decides to procrastinate*. Although one may come to regret the decision to procrastinate later, at the time the decision to procrastinate was made, one could argue that it was an intentional decision to put off completing the required task. Alternatively, when one procrastinates, one does not consider the likely negative effects of procrastination. It is not always a choice but it may be an impulsive decision to engage in the alternative activity. This realization may identify one means of dealing with both procrastination and the impulsive choice of the smaller sooner reward. If one understands the appeal of the smaller sooner reward, one can plan ahead to avoid putting oneself in a position to make that choice. For example, to avoid the “temptation” to socialize with friends or watch TV, a student may decide to go to the library to begin working on a term paper. One can think of going to the library as making a *prior commitment* to work on the paper. Ariely and Wertenbroch (2002) found that people are willing to create self-imposed deadlines to help overcome procrastination.

**Figure 2:**
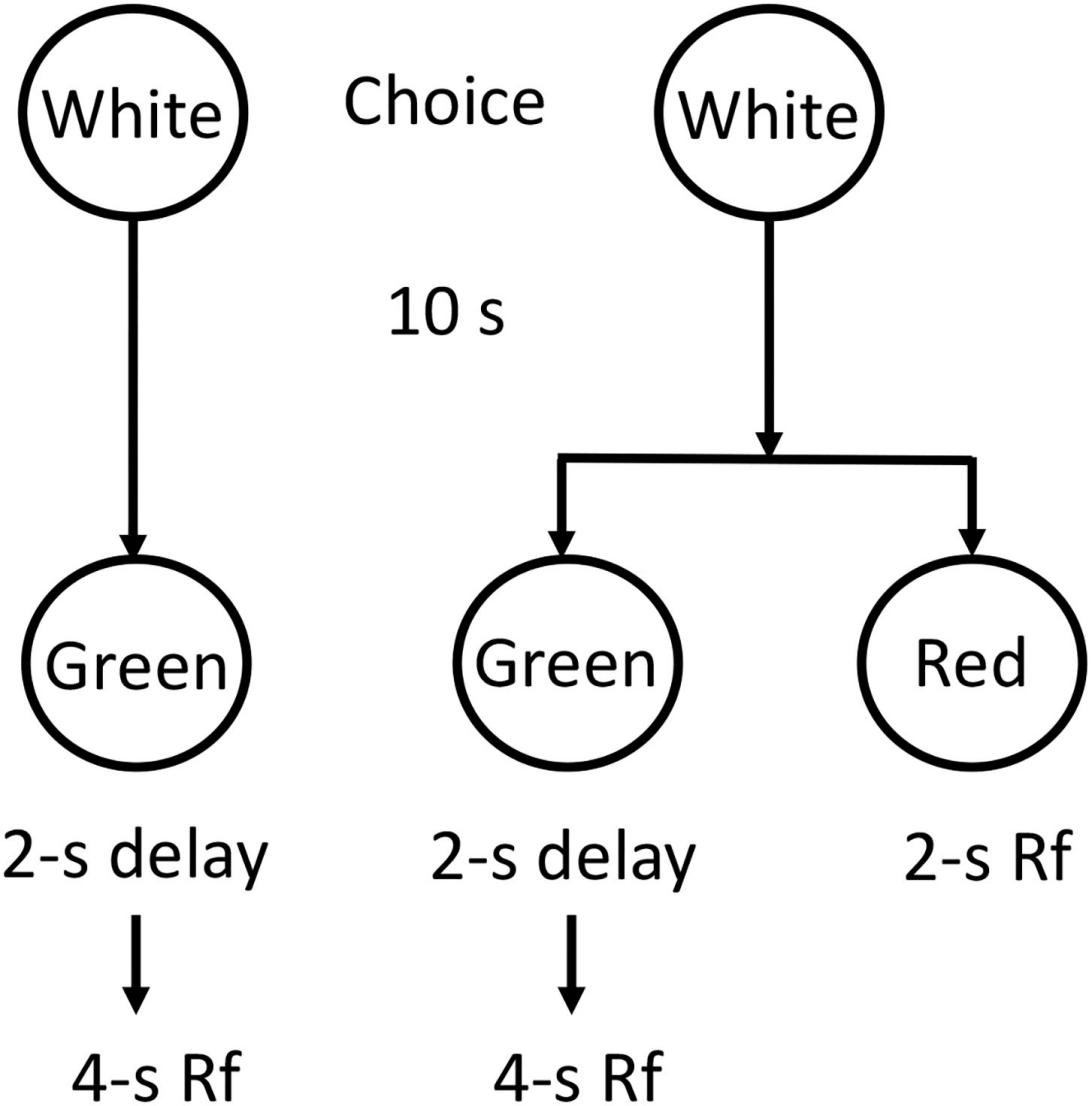
Design of the Rachlin and Green (1972)experiment. Given an immediate choice between Green (2-s delay and 4-s reinforcement) and Red (immediate 2-s reinforcement) pigeons preferred Red. However, given an initial choice between Green alone or a choice between Green and Red, pigeons preferred Green alone. It looks like they make a “commitment” to avoid the “temptation” to choose Red.

Surprisingly, a similar effect can be demonstrated in non-human animals. The concept of making a prior commitment was tested in pigeons by Rachlin and Green (1972). They first showed that for a given choice between a smaller sooner reward and a (two times) larger later reward, the delay was such that the pigeons preferred the smaller sooner reward. They then modified the procedure such that 10 s prior to being given the choice between the smaller-sooner/larger-later reward, the pigeons were given a choice between receiving the delayed smaller-sooner/larger-later choice and making a commitment to receive only the larger-later outcome (see Figure 2). That is, they could choose to later choose between the smaller sooner and larger later or choose to commit to the larger later. When given this earlier choice, the pigeons elected to avoid the later smaller-sooner/larger-later choice and instead they chose to commit themselves to the larger later reward. Thus, getting the pigeons to make a prior commitment got them to make the optimal choice. For the student needing to write the term paper, the decision to go to the library to work on her term paper before encountering the immediacy of alternative pleasurable activities, likely serves a similar function.

### Nature of the Function: Exponential With 2 Different k Values or Hyperbolic With Only One

The decline in the delay discounting function with time to reinforcement can be described either by an exponential decay function or a hyperbolic decay function. The exponential (e) would take the form of Equation 1 (Mazur, 1987) in which V = the value of the discounted reward, A = the magnitude of the reward, D = the delay to the reward, and *k* = a constant that represents the rate at which the value declines with delay.


(1) $$\begin{array}{l} {V=\;Ae}^{-kD} \end{array}$$

The hyperbolic decay function would take the form of Equation 2 (Mazur, 1987).


(2) $$\begin{array}{l} V=\;\frac{A}{1+kD} \end{array}$$

In this equation, as well, V = the value of the discounted reward, A = the magnitude of the reward, D = the delay to the reward, and k = represents the rate at which the value declines with delay.

**Figure 3:**
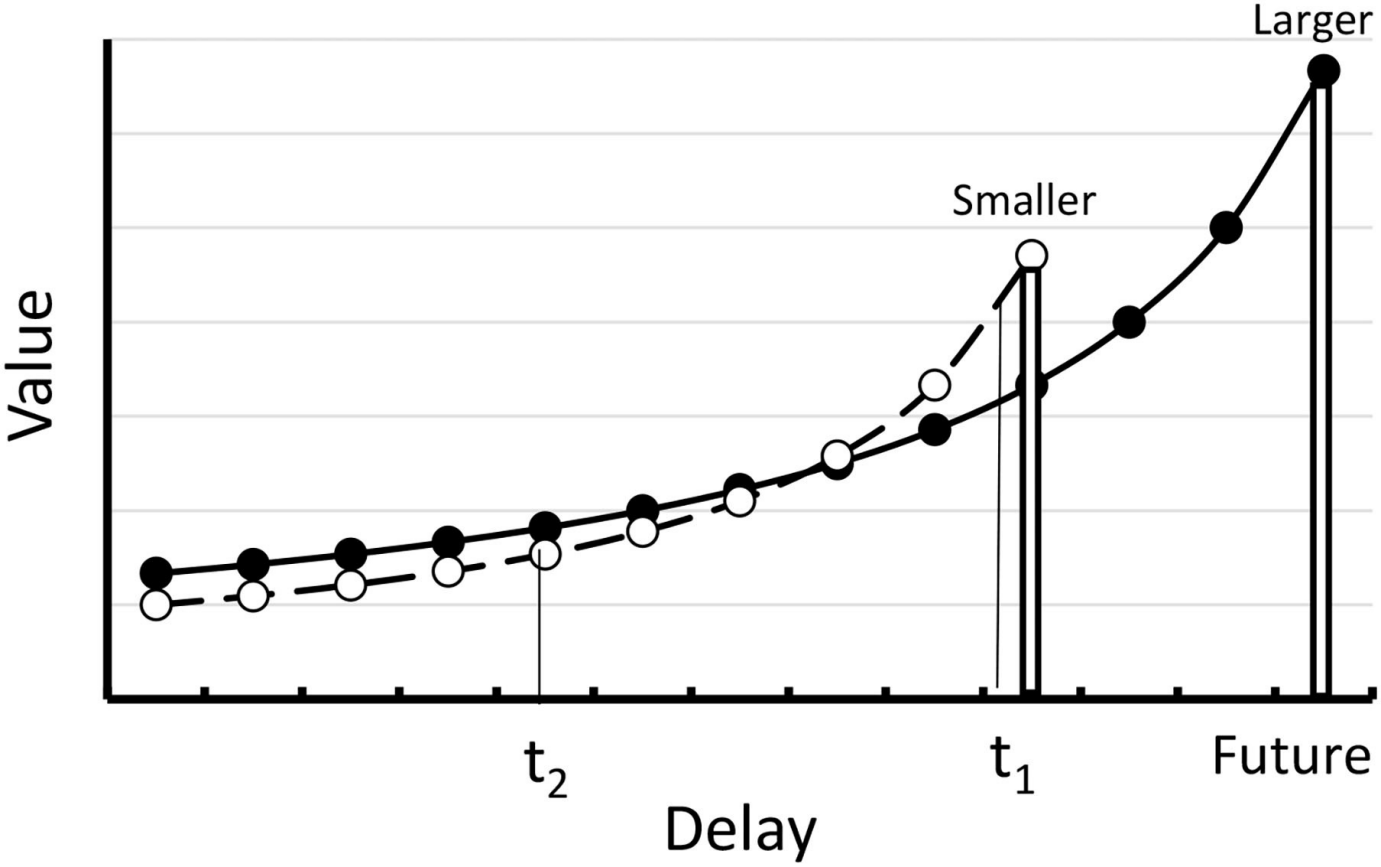
Hyperbolic delay discountingfunction. At time 1 (t_1_) the value of the smaller reward is greater than the value of the larger reward. At time 2 (t_2_) there is a reversal of preference.

The exponential conforms empirically to the preference for the smaller sooner over the larger later reward, but it does not account for the well documented reversal of preference when equal increments in delay are added to both the smaller and larger rewards (Rachlin and Green, 1972; Ainslie, 1974; see Figure 3). The exponential decay function can be salvaged if one assigns greater *k* value to the smaller sooner reward than to the larger later reward, but that requires two different values to the *k* parameter.

The hyperbolic delay function (see Figure 3) allows for the reversal of preference without assuming two different values of *k*. According to the hyperbolic decay function, assuming that at time *t*_1_ one prefers the smaller sooner reward over the larger later reward, the preference can reverse if the choice between the smaller sooner and larger later can be made somewhat earlier (e.g., at *t*_2_ rather than at *t*_1_). Thus, the hyperbolic function can explain how it might be that before the choice between the task and competing activities are immanent, at a time when one has the intention of start to work on the task at an appropriate time, one can make a commitment to not be tempted by other activities. According to the hyperbolic delay discounting function, in the absence of such a commitment, when other activities become more immediate, they will take on more value and will complete more successfully with the larger later task.

## The Free Operant, Temporal Avoidance Procedure

Delay discounting is the basis of Steel and König (2006) temporal motivation theory. It is based on the discounting of rewards that will occur in the future. The weakness of delay discounting as an account of procrastination is it does not capture the build-up in anxiety that often accompanies procrastination because the in the typical delay discounting procedure, the delayed event is not aversive. A better analog of procrastination that does involve a future aversive task is the free operant, temporal avoidance procedure (Sidman, 1966, often referred to as Sidman avoidance). With this procedure, for example, a rat is given a shock unless it makes a response (e.g., jumping over a barrier) and it must do so, for example, within 20 s of its last response (or shock). In spite of the fact that there is no external stimulus presented to alert the rat to the impending shock, rats readily learn to jump over a barrier to avoid the shock. An interesting problem for learning theory is, given that a rat learns to successfully avoid the shock, it does so for a very long time. But what maintains the response in the absence of an occasional shock to support the jumping response (see Solomon et al., 1952).

According to Mowrer's (1947) two-factor theory, in avoidance learning there are two factors. The first factor is the Pavlovian association that develops between the signal for shock (in this case the passage of time) and the shock. Due to that association, the passage of time following the last response or shock is presumed to elicit fear. The animal then learns that the cues (feedback) from making the instrumental response (the jump) become safety signals for the absence of shock (see Bolles and Grossen, 1969). Those safety signals result in fear reduction, negative reinforcers. Thus, in Sidman avoidance, although there is no external signal for shock, the passage of time since the last response or shock may serve such a function.

If fear of shock in the free operant temporal avoidance procedure is analogous to fear (or anxiety) associated with missing the deadline for humans, one might expect to see a form of procrastination by the rats as they learn about the contingencies of the task. In the temporal avoidance procedure, to avoid getting shocked, the rats can jump over the barrier at any time before the shock. Once they have learned the contingencies of the task, a temporal discrimination, however, they tend not to press the lever immediately following the last response or shock. Instead, they generally delay making the shock avoidance response until near the end of the interval (Sidman, 1962; Hineline and Herrnstein, 1970). That is, they tend to procrastinate by waiting until shortly before the shock would arrive.

According to two-factor theory, the passage of time increases fear, but with training, the rats learn that shock will not come early in the interval, so they are not fearful, and they do not press earlier than they need to. The fear presumably builds up gradually until it reaches the point at which the shock is soon expected, and then the response is made.

**Figure 4:**
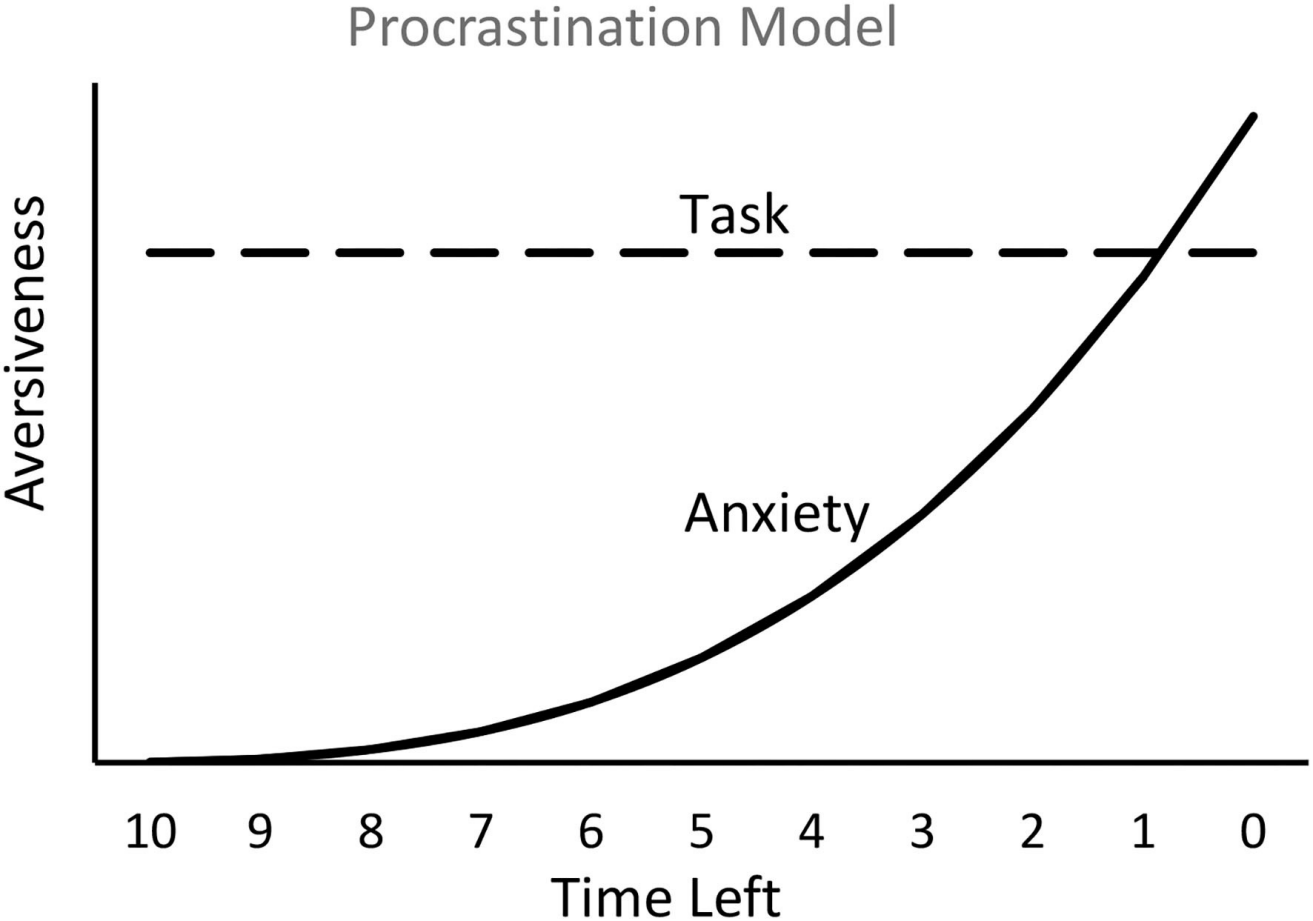
Hypothetical plot of the relation between the interaction of two emotions, the aversiveness of the task and the aversiveness of anxiety resulting from the fear of doing poorly or of missing the deadline. The model predicts that work on the task will begin when anxiety approaches the aversion to the task.

A simplified model of human procrastination is assumed to follow a time course similar to that of the temporal avoidance procedure. The aversiveness of the task is relatively constant, whereas the anxiety (presumably resulting from the fear of not completing the task prior to the deadline) builds up, gradually at first and then progressively faster (see Figure 4). The model presented in Figure 4 assumes that the source of procrastination is competition between two aversive motivations, task aversiveness and anxiety. From a learning theoretic perspective, however, one might expect that the many experiences of building anxiety that one undergoes, over many instances of task postponement, would result in reduced procrastination. In fact, people who procrastinate often do have regrets (Ferrari et al., 2009), and they often assert that in the future they will start working on the required task earlier—a resolution that often goes unfulfilled. A possible explanation for the maintenance of procrastination, even with continued feelings of frustration at the build-up of anxiety each time, is that the procrastination behavior itself may be reinforced. I will now address that possibility.

## Recent Animal Research

### Can Task Completion Shortly Before a Deadline Serve as a Reinforcing Event?

**Figure 5:**
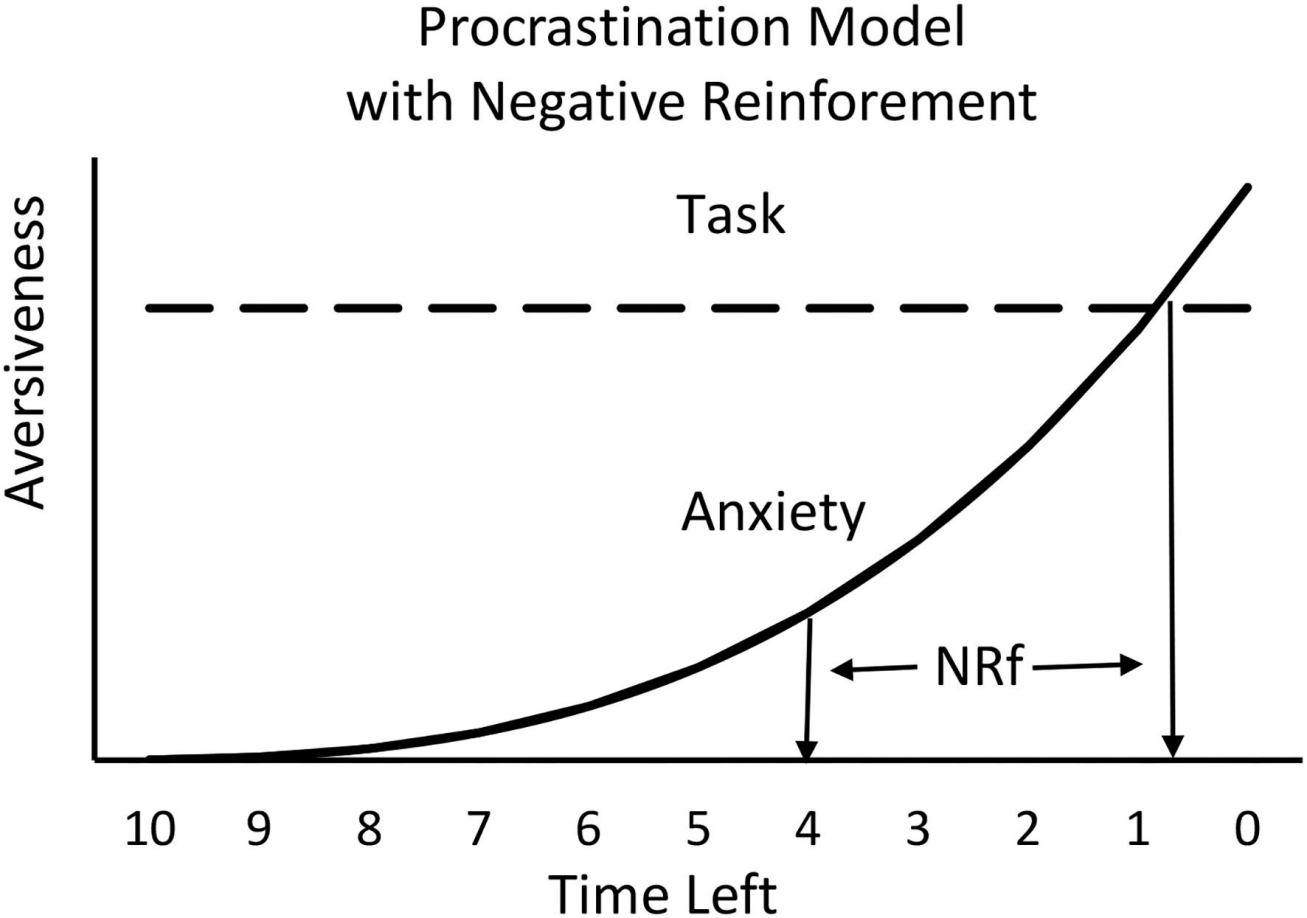
Negative reinforcement (NRf) associated with the reduction in anxiety will depend on the amount of anxiety experienced and will increase as the time left approaches the deadline.

As noted earlier, people who procrastinate often vow that in the future they will start working on the required task earlier. Yet, chronic procrastinators rarely do. To account for persistent procrastination, one can posit that there is something about procrastination itself that is reinforcing. If procrastination produces an increase in anxiety (a negative affect), a reasonable source of reinforcement might be negative reinforcement in the form of relief (the removal of an aversive event) when the task has been completed before the deadline. Furthermore, the closer to the deadline the task is completed, the greater would be the anxiety prior to working on the task and thus, the greater the magnitude of the negative reinforcer would be upon task completion. For this reason, it is possible that the sense of relief that one experiences when completing a task close to the deadline actually maintains procrastination. A model of this negative reinforcement effect appears in Figure 5, a modification of Figure 4.

To better understand the negative reinforcement that may contribute to procrastination, it may be useful to consider the stimulus events that take place, independent of whether the task outcome is negative or positive reinforcement and relate those events to basic principles of reinforcement.

Fantino's (1969) delay reduction theory provides a useful foundation. According to delay reduction theory, signals for reinforcement become stimuli associated with reinforcement (conditioned stimuli) to the extent that they predict reinforcement better when they are present than when they are absent. This appears to be a simple premise, but it makes some important predictions. According to this theory, it is not only how close in time the signal is to reinforcement, but it is how close in time the signal is to reinforcement, *relative to other events*. A nonobvious prediction of delay reduction theory is, given that a stimulus occurs at a fixed time from reinforcement, the longer the interval *prior* to the onset of that stimulus, the better a conditioned stimulus it will become (Fantino, 1969). That is, it is the *relative* proximity to the reinforcer rather than its absolute proximity that is crucial. Thus, the longer the interval between a given stimulus-reinforcer pairing, the more effective the conditioned stimulus should become. Gibbon et al. (1977; see also Singer et al., 2007) have found support for this prediction.

To better understand this concept, imagine that the pairing of a light with food occurs every 10 s. In this case, it should be relatively easy to predict the occurrence of food, even in the absence of the light. If the pairing of the light with the food occurred once every hour, however, it would be relatively difficult to predict the food in the absence of the light. Thus, the light would become a better signal for the occurrence of food.

Before seeing how this theory may apply to procrastination, we decided to test delay reduction theory in a context more relevant to procrastination than had been done earlier. That is, with a procedure in which the total time to reinforcement (the “deadline”) is held constant, but the duration of conditioned stimulus associated with the deadline is varied.

**Figure 6:**
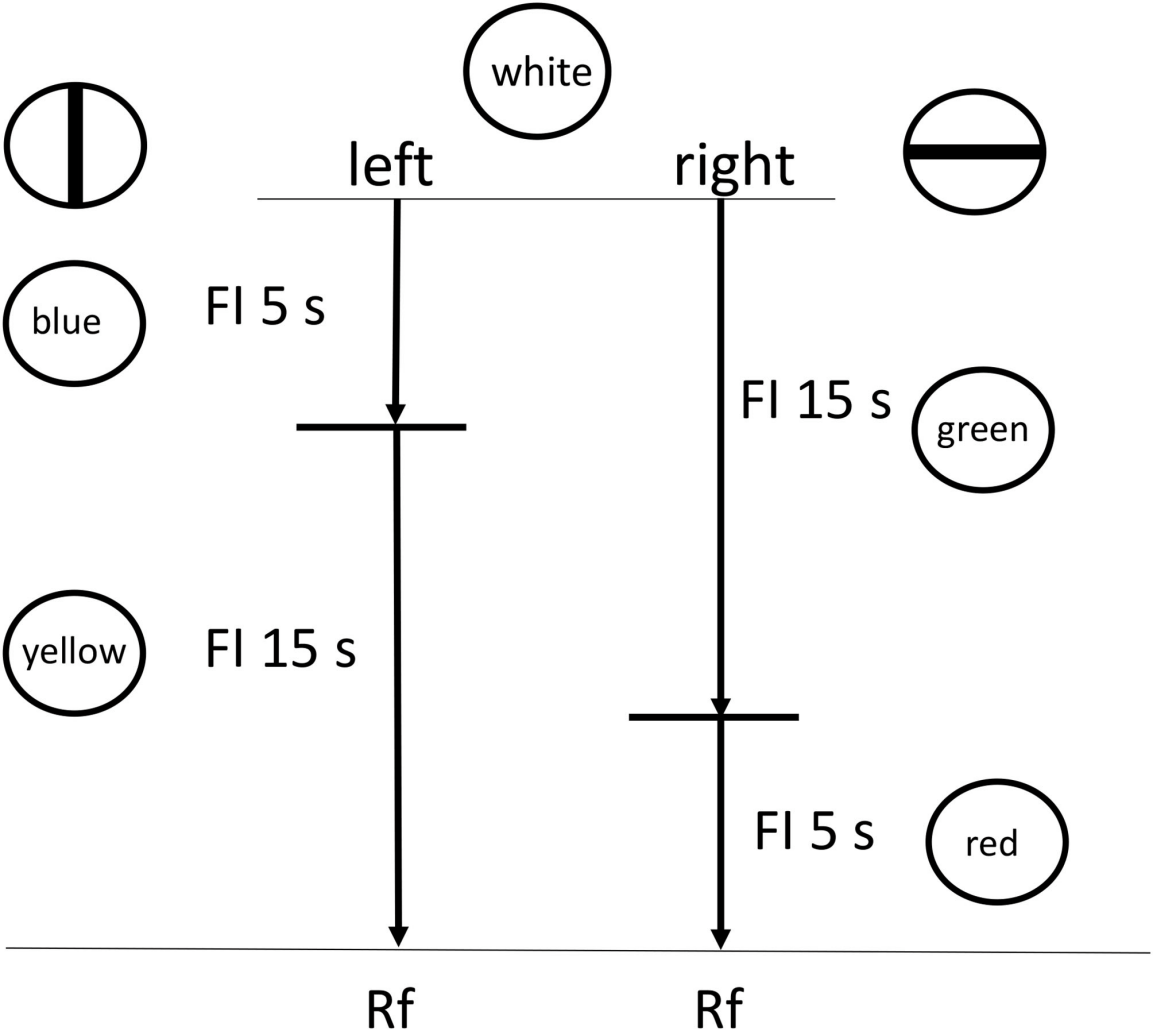
Design of the Zentall et al. (2018) experiment. Pigeons were given a choice between two reinforced chains, short (FI 5 s) followed by long (FI 15 s) or long (FI 15 s) followed by short (FI 5 s).

Imagine the following scenario: there is a fixed delay to reinforcement (or in the case of procrastination, the deadline) with a choice between two stimulus chains (to procrastinate or not). One chain starts with presentation of a long interval, signaled by Stimulus A (analogous to the period of procrastination, before working on the task), followed by a short interval, signaled by Stimulus B (analogous to time working on the task), followed by reinforcement (see Figure 6). The other chain starts with presentation of a short interval, signaled by Stimulus C (time working on the task), followed by a long interval, signaled by Stimulus D (time after working on the task), followed by reinforcement. At this point there is no actual task, just stimuli that signal time periods prior to the reinforcer.

According to delay reduction theory, although the two chains represent the same total delay to reinforcement, the chain represented by the long interval followed by the short interval presents a short interval just prior to reinforcement. Thus, that stimulus should serve as a very good, conditioned stimulus. Not only should the short Stimulus B be preferred over the long Stimulus D that also appears just prior to the reinforcer, but importantly, the short Stimulus B also should be preferred over the short Stimulus C that occurs earlier in the other chain (Fantino, 1969). If presentation of the long interval early can be thought of as analogous to deferring task completion, and presentation of the short stimulus early as analogous to completing the task early, could experiencing the short stimulus immediately before the reinforcer (the deadline) be more reinforcing than experiencing the short stimulus earlier?

**Figure 7:**
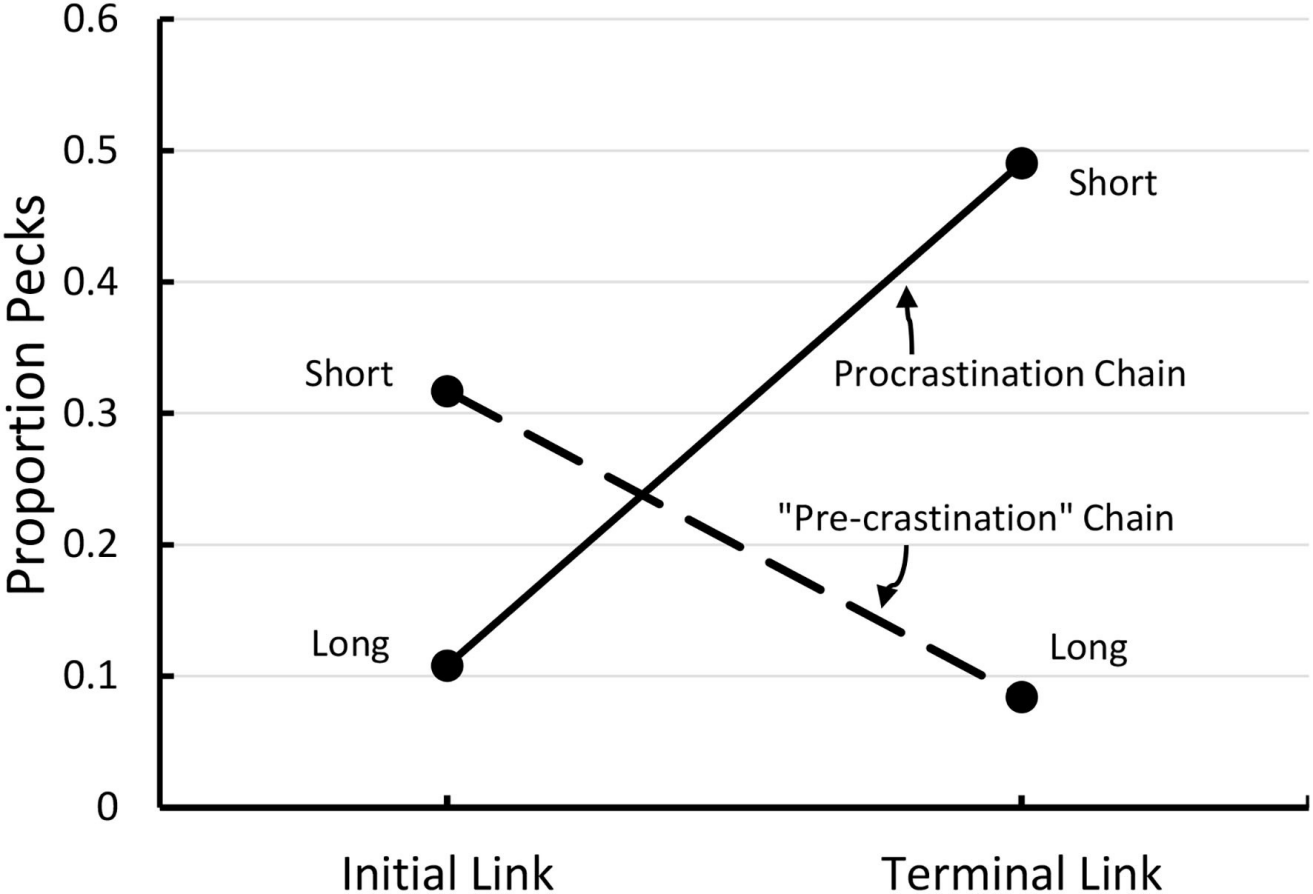
Proportion of pecks to each of the four components of the two chains depicted in Figure 5. Pigeons preferred the long-short (procrastination) chain over the short-long (“pre-crastination”) chain.

Initially, we tested this hypothesis by asking if pigeons would prefer a long-short interval chain over a short-long interval chain, both of the same total duration. In our first experiment (see Figure 6), the pigeons showed a 2–1 preference for the long-short interval chain over the short-long interval chain (Zentall et al., 2018). In addition, the relative peck rate to the short 5 s stimulus that was immediately followed by reinforcement (Stimulus B) was significantly greater than to both the short 5 s stimulus (C) that was followed by the relatively long 15 s stimulus (D), and to the relatively long 15-s stimulus (D) that was immediately followed by reinforcement (see Figure 7). Thus, it appears that the short stimulus that was followed by reinforcement became a stronger conditioned stimulus, and it is likely that it was responsible for the preference for the long-short interval chain.

Although this experiment did not include an aversive event or a task that one would normally associate with procrastination, it did show that task completion events close to the deadline may become strong conditioned stimuli. The next step was to interpolate a mildly aversive event between the two intervals in each chain to simulate a task that elicits the motivation to defer.

Given the nature of the concurrent chains task, we were looking for an event that had been shown to be mildly aversive, one that might be seen as analogous to writing a term paper. McDevitt et al. (1997) found that preference for a conditioned stimulus was greatly reduced when a period with no stimulus, a dark period preceded it, even when the dark period did not increase the time to reinforcement. If one views the dark period as a mildly aversive event, one can ask, if pigeons are not able to avoid a dark period, would they choose to defer the dark period to later in the trial?

**Figure 8:**
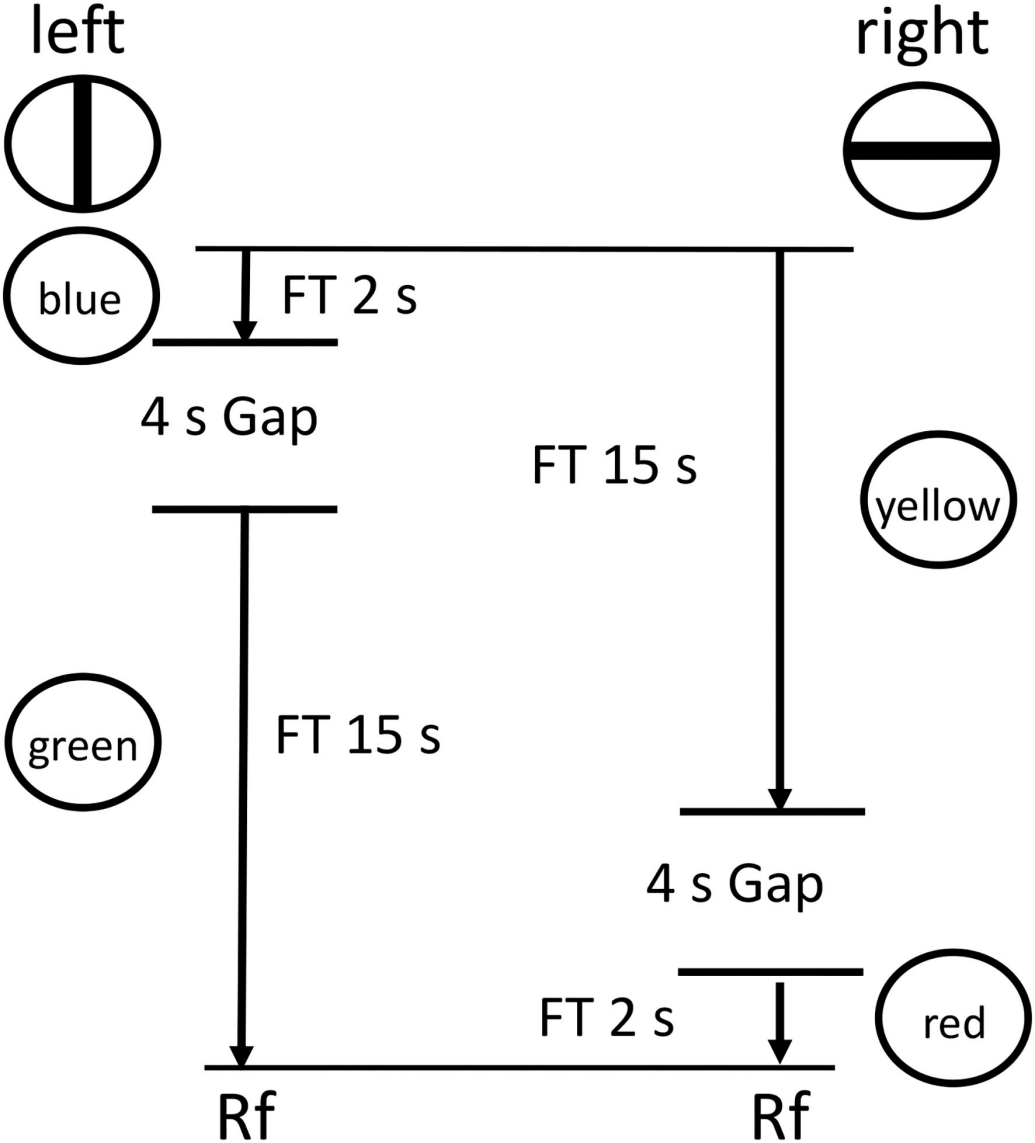
Design of the Zentall et al. (2020, Experiment 1) experiment. Pigeons were given a choice between two reinforced chains, short (FI 2 s) followed by long (FI 15 s) or long (FI 15 s) followed by short (FI 2 s), with a 5 s dark period (a gap) between the two links of each chain.

Zentall et al. (2020, Experiment 1) tested pigeons with a procedure similar to that used by Zentall et al. (2018) but they added a 4 s dark period between the two intervals in each chain (see Figure 8). They found, once again, that pigeons chose to delay experiencing the relatively aversive dark period. That is, they procrastinated receiving the aversive dark period.

**Figure 9:**
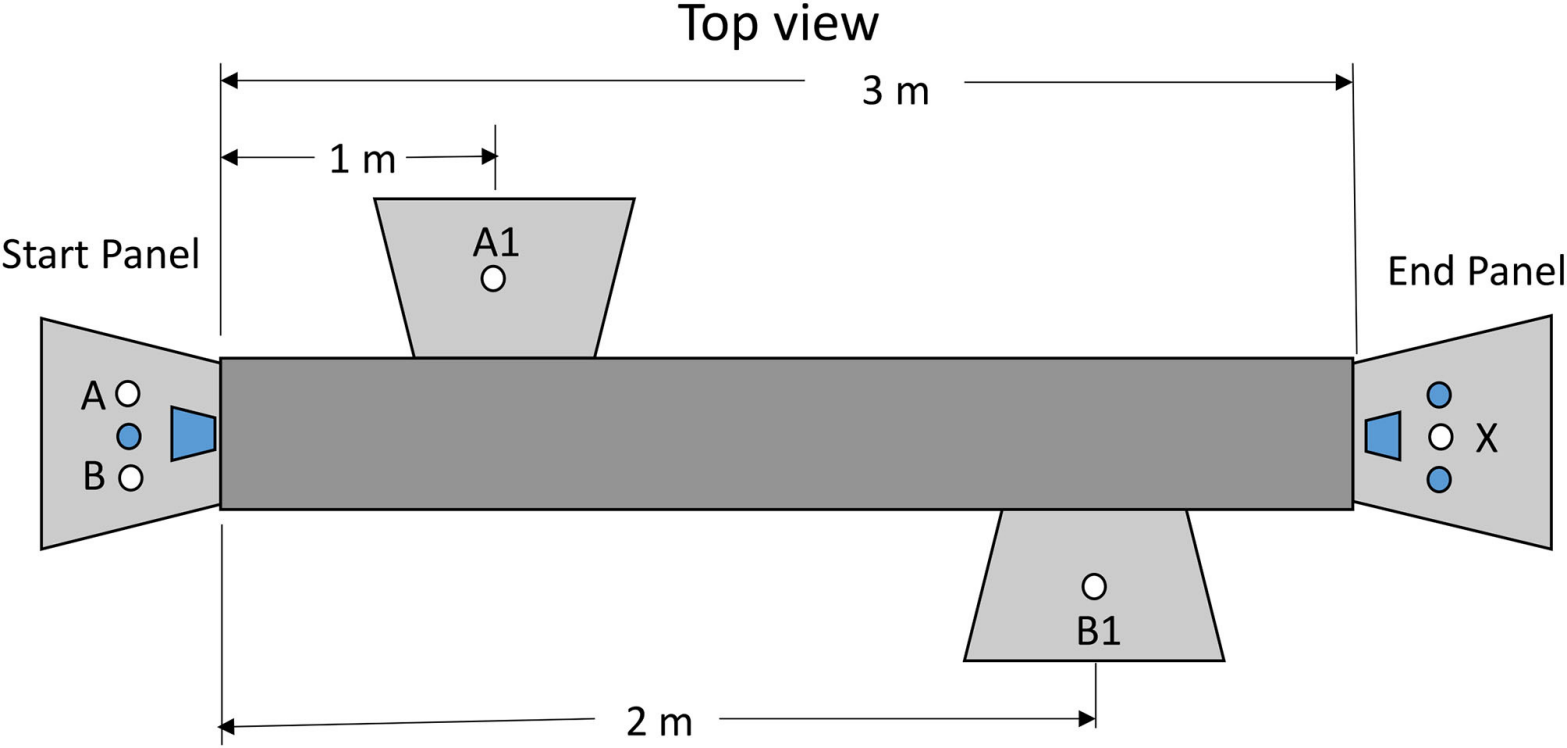
Apparatus used in the Zentall et al. (2020, Experiment 1). Pigeons made a choice between stimulus lights A and B. If they chose A, they would have to peck A1 10 times and then peck stimulus X for reinforcement. If they chose B, they would have to peck B1 10 times and then peck stimulus X for reinforcement.

Although we now had suggestive evidence that pigeons would defer a relatively aversive dark period in a chain that led to reinforcement, we wanted to test pigeons with a task that more closely resembled a human procrastination task. For that purpose, we selected a task in which to obtain a reinforcer, the pigeon had to walk from one end of a long cage to the other end of the cage (see Figure 9). On the way, the pigeon had to perform a pecking task (Zentall et al., 2020, Experiment 2). The pecking could occur closer to the start or closer to the goal (farther from the start). The results of this experiment indicated that the pigeons preferred to defer the peck requirement. That is, they preferred to procrastinate making the side-key pecking response.

Taken together, the results of the pigeon research are consistent with the hypothesis that deferring a task requirement can be reinforcing for pigeons. For humans, if one considers the relief that one feels upon completion of a task as serving as a negative reinforcer, and the closer task completion comes to the deadline, the greater the negative reinforcer (the removal of an aversive event), it can potentially explain why it is that procrastination can become a habitual behavior. Although one may readily remember the build-up of anxiety that occurs as the deadline approaches, and that build-up of anxiety may be responsible for a pledge to avoid procrastinating in the future, it may well be that it is the relief from anxiety that occurs upon completion of the task that makes it difficult to stick to one's intention not to procrastinate in the future.

## Conclusions

The ubiquity of procrastination suggests that certain basic behavioral processes are likely to be involved. The fact that these basic behavioral processes demonstrate behavior similar to human procrastination suggests that cultural factors are not likely to be necessary and individual differences such as laziness, fear of failure and perfectionism are not critical components. Temporal motivation theory proposed by Steel (2007) demonstrates the role that delay discounting plays in procrastination and that phenomenon has been widely demonstrated in humans and other animals (e.g., Ainslie, 1974). In addition, it is very likely that free-operant temporal avoidance learning (Sidman, 1966), delay reduction theory (Fantino, 1969), and negative reinforcement (Zentall et al., 2020) all contribute to procrastination and especially to its persistence. Although some researchers have viewed procrastination as a trait rather than as behavior that is context and experience dependent, the involvement of these basic mechanisms suggests that people may learn to procrastinate. Furthermore, if negative reinforcement, associated with the abrupt decline in anxiety that typically accompanies task completion, reinforces procrastination, it may be difficult for one to avoid procrastinating in the future, even if one has the best of intentions. The demonstration of the similarity of the behavior of non-human species to human procrastination suggests, importantly, that cultural factors as well as human traits such as perfectionism, laziness, and fear of failure are not necessary to explainhuman procrastination.

## Data Availability Statement

Publicly available datasets wereanalyzed in this study. These data can be obtained from the author at zentall@uky.edu.

## Ethics Statement

The animal studies reported here were reviewed and approved by the University of Kentucky Institutional Animal Care and Use Committee.

## Author Contributions

The author confirms being the sole contributor of thiswork and has approved it for publication.

## Conflict of Interest

The author declares that the research was conducted in the absence of any commercialor financial relationships that could be construed as a potential conflict of interest.

## Publisher's Note

All claims expressed in this article are solely those of the authors and do not necessarily represent those of their affiliated organizations, or those of the publisher, the editors and the reviewers. Any product that may be evaluated in this article, or claim that may be made by its manufacturer, is not guaranteed or endorsed by the publisher.
